# *Paracoccidoides brasiliensis* 30 kDa Adhesin: Identification as a 14-3-3 Protein, Cloning and Subcellular Localization in Infection Models

**DOI:** 10.1371/journal.pone.0062533

**Published:** 2013-04-30

**Authors:** Julhiany de Fatima da Silva, Haroldo César de Oliveira, Caroline Maria Marcos, Rosângela Aparecida Moraes da Silva, Tania Alves da Costa, Vera Lucia García Calich, Ana Marisa Fusco Almeida, Maria José Soares Mendes-Giannini

**Affiliations:** 1 Department of Clinical Analyses, Faculty of Pharmaceutical Sciences, São Paulo State University - University Estadual Paulista Araraquara, São Paulo, Brazil; 2 Department of Immunology, Biomedical Institute, São Paulo University, São Paulo, Brazil; Leibniz Institute for Natural Products Research and Infection Biology- Hans Knoell Institute, Germany

## Abstract

*Paracoccidoides brasiliensis* adhesion to lung epithelial cells is considered an essential event for the establishment of infection and different proteins participate in this process. One of these proteins is a 30 kDa adhesin, pI 4.9 that was described as a laminin ligand in previous studies, and it was more highly expressed in more virulent *P. brasiliensis* isolates. This protein may contribute to the virulence of this important fungal pathogen. Using Edman degradation and mass spectrometry analysis, this 30 kDa adhesin was identified as a 14-3-3 protein. These proteins are a conserved group of small acidic proteins involved in a variety of processes in eukaryotic organisms. However, the exact function of these proteins in some processes remains unknown. Thus, the goal of the present study was to characterize the role of this protein during the interaction between the fungus and its host. To achieve this goal, we cloned, expressed the 14-3-3 protein in a heterologous system and determined its subcellular localization in *in vitro* and *in vivo* infection models. Immunocytochemical analysis revealed the ubiquitous distribution of this protein in the yeast form of *P. brasiliensis*, with some concentration in the cytoplasm. Additionally, this 14-3-3 protein was also present in *P. brasiliensis* cells at the sites of infection in C57BL/6 mice intratracheally infected with *P. brasiliensis* yeast cells for 72 h (acute infections) and 30 days (chronic infection). An apparent increase in the levels of the 14-3-3 protein in the cell wall of the fungus was also noted during the interaction between *P. brasiliensis* and A549 cells, suggesting that this protein may be involved in host-parasite interactions, since inhibition assays with the protein and this antibody decreased *P. brasiliensis* adhesion to A549 epithelial cells. Our data may lead to a better understanding of *P. brasiliensis* interactions with host tissues and paracoccidioidomycosis pathogenesis.

## Introduction

*Paracoccidoides brasiliensis* is a dimorphic fungus and the etiologic agent of paracoccidioidomycosis (PCM). This disease presents prolonged evolution and may involve several organs [1]. *P. brasiliensis* is considered a facultative intracellular fungus that can adhere to and invade epithelial cells *in vivo* and *in vitro* [2]. The adhesion and invasion abilities of the fungus are dependent on the virulence of the isolate [3], which can be attenuated or lost after subsequent cycles of subculture for long periods [4] and reestablished after passage in animals [5] or in epithelial cell culture. *P. brasiliensis* has multiple mechanisms of pathogenicity, including adherence, colonization, dissemination, survival in hostile environments and escape from immune response mechanisms that allow it to colonize the host and cause disease [6]–[8]. The fungus also uses a variety of surface molecules to bind to the extracellular matrix of the host cell and establish infection [9]. The molecular mechanisms involved from first contact with the infectious agent to subsequent stages of the disease remain unknown. A necessary step in the colonization and, ultimately, development of diseases by pathogens is associated with their ability to adhere to the surface of the host. The ability to adhere is a widely distributed biological phenomenon that is shared by many organisms to enable them to colonize their habitats. Successful colonization is usually a complex event and involves surface proteins of the fungus and cellular receptors [10], [11]. In this way, PCM development depends on interactions between the fungus and the host cell components.

Fungal virulence is a highly complex event resulting in the expression of multiple genes at different stages of infection, and adhesion and survival of the pathogen within the host appear to be essential in establishing pathogenesis. In this context, important virulence factors of the fungi have been described [2], [12]–[19]. Pathogen adhesion requires the recognition of carbohydrate or protein ligands on the surface of the host cell or proteins of the extracellular matrix (ECM) [20]–[22]. Studies have characterized extracellular matrix components involved in the interaction between *P. brasiliensis* and the host, and some adhesins have also been described. Adhesins are believed to play an important role in *P. brasiliensis* pathogenesis [3], [23]–[35].

The large number of different tissues that fungi can colonize and infect suggests that fungi can use a variety of surface molecules for adhesion [36]. Mechanisms that may be responsible for determining the pathogenicity and virulence of *P. brasiliensis* have been extensively investigated by interaction experiments of this pathogen *ex vivo* in cell culture [26], [27], [37]–[42] and experiments using high-throughput molecular tools, such as cDNA microarrays, insertion and/or gene deletion, and RNA interference [14], [43]–[50]. Studies have characterized extracellular matrix components involved in the interaction of *P. brasiliensis* with the host. The ECM consists of a network of proteins, including collagen, non-collagen glycoproteins, especially fibronectin and laminin, and proteoglycans, which seem to affect the proliferative capacity of the fungus [2]. In general, genes involved in adhesion are not constitutively expressed but activated when induced at the site of infection in the host [51], [52]. The understanding and identification of molecules involved in the adhesion of microorganisms to different substrates in the host are important as targets for more effective new treatments in systemic mycoses.

Some molecules of *P. brasiliensis* have been identified as ligands of extracellular matrix components. Gp43 was the first to be identified as a ligand for laminin [3], [23], [24]. The 43 kDa glycoprotein was found to play a role in adhesion because anti-gp43 serum inhibited the adhesion process by 85% [3]. Additional tests of binding affinity showed that gp43 was able to bind both fibronectin and laminin. In *P. brasiliensis*, other adhesins have also been described, and they are believed to play important roles in its pathogenesis [26], [27], [29], [32]–[35], [39], [53]. A 30 kDa adhesin of *P. brasiliensis*, which is capable of binding to laminin, was isolated and found to be expressed at higher levels in a *P. brasiliensis* isolate that showed high adhesion capacity [39]. *P. brasiliensis* also presents two proteins on its cell surface with molecular weights of 19 and 32 kDa that interact with different ECM proteins, including laminin, fibrinogen and fibronectin. Assays using conidia of *P. brasiliensis* pre-incubated with anti-32 kDa monoclonal antibody inhibited the adhesion of fungal proteins to the ECM in a dose-dependent manner [29], [54]. Recently, protein sequence analysis characterized the 32 kDa as a hydrolase, and knockout mutants showed changes in morphology, a reduced ability to adhere to human epithelial cells *in vitro* and decreased virulence in infection models in mice [31], [54]. In addition to these adhesins, enzymes of *P. brasiliensis* that interact with host molecules are regarded as adhesin-like, such as GAPDH (glucose-6-phosphate dehydrogenase), a ligand of laminin, fibronectin and collagen type I [26], TPI (triosephosphate isomerase), which also binds to matrix components, such as laminin and fibronectin [34], and ICL (isocitrate lyase), a ligand of laminin, fibronectin and collagen type I [55], [56]. Additionally, malate synthase (MLS) of *P. brasiliensis*, which functions in the glyoxylate cycle and allantoin pathway, is located in the cytoplasm and the surface, especially in budding cells. This protein is secreted and acts as an adhesin, indicating its multifunctional role [33]. Da Silva Castro et al., (2008) [57] described another fungal surface molecule, called PbDfg5p, that has the capacity to adhere to ECM proteins. This protein was characterized as belonging to the family of glycosyl hydrolases and is related to the formation and maintenance of the fungal cell wall. In *P. brasiliensis*, its presence was detected in the cell wall and cell wall protein extracts obtained from yeast treated with β-1-3 endoglucanase using electron microscopy and immunogold labeling. Recombinant PbDfg5p displayed an ability to bind to laminin, fibronectin, collagen type I and type IV and contained an RGD motif (Arg-Gly-Asp, which binds to fibronectin) in its predicted sequence, a common characteristic of some adhesins [57].

In our study, this 30 kDa adhesin was identified as a 14-3-3 protein using Edman degradation and mass spectrometry analysis. The 14-3-3 protein family is a highly conserved group of small acidic proteins that have been implicated in a variety of cellular processes in eukaryotes. However, although these proteins are involved in apoptosis, signal transduction, cell cycle regulation and transcription, their exact role in these processes remains unknown [58]. Members of this group function as accessory proteins in various processes, act as specific determinants that alter the cellular localization of other proteins with which they interact and are involved in the direct regulation of enzyme activity [59].

Thus, in this study, we characterized the 14-3-3 protein of *P. brasiliensis* by determining its localization, both in the yeast form of the fungus and in infection models (epithelial cells and a murine model), to better understand *P. brasiliensis-*host tissue interactions and paracoccidioidomycosis pathogenesis.

## Materials and Methods

### Ethics Statement

Animal experiments were performed in strict accordance with Brazilian Federal Law 11,794 establishing procedures for the scientific use of animals and the state law establishing the Animal Protection Code of the State of São Paulo. All efforts were made to minimize suffering, and all animal procedures were approved by the Ethics Committee on Animal Experiments of the Institute of Biomedical Sciences of the University of São Paulo (Proc.180/2011/CEUA) and the Ethics Committee on Animal Experiments of the Faculty of Pharmaceutical Sciences of Araraquara – UNESP (Proc. 10/2011/CEUA/FCF).

### *P. brasiliensis* Isolate and Growth Conditions

A highly virulent *P. brasiliensis* (isolate 18), obtained from the mycology collection of the Faculty of Medicine, University of São Paulo (FM-USP), was used throughout this investigation. *P. brasiliensis* yeast cells were maintained by weekly subcultivation in semisolid culture medium. Fungal cells were grown for 3–4 days at 35°C on Fava-Netto solid medium [60].

### Protein Characterization by Amino Acid Sequencing

For internal peptide sequencing, the 30 kDa protein was subjected to two-dimensional electrophoresis. The gel was stained with Coomassie blue, and the band was excised from the gel, eluted, and digested with trypsin for endopeptidase digestion. The fragments were separated by reverse-phase HPLC and subjected to Edman degradation [61].

### Amino Acid Sequence Homology Analysis of *P. brasiliensis* 30 kDa Adhesin

The amino acid sequences were compared to other sequences deposited in a database. The homology searches were performed with the BLASTP program [62] and FASTA 3 [63].

### Cloning cDNA Containing the Complete Coding Region of the 14-3-3 Protein into an Expression Vector

Cloned cDNA containing the complete coding region of the 14-3-3 protein (GenBank accession number AY462124) [64] was amplified by PCR using sense (5′-ATGGGTTACGAAGATGCTG-3′) and antisense (5′-CTCAGCGGCCTTAGGAGC-3′) primers. The amplification parameters were as follows: an initial denaturation step at 94°C for 2 min, followed by 25 cycles of denaturation at 94°C for 30 s, annealing at 58°C for 30 s, and extension at 72°C for 1 min and 10 s. A final elongation step was performed at 72°C for 7 min. The PCR product was subcloned into the *Sal*I/*Xho*I sites of the pET-32a(+) expression vector (Novagen, Inc., Madison, WI, USA.). The resulting plasmid was transformed into *Escherichia coli* DH10B. Bacteria transformed with pET-32a-14-3-3r were grown in LB medium supplemented with ampicillin (100 µg/mL) at 37°C to an optical density of 0.6 at 600 nm. Recombinant protein production was induced by adding 0.4 mM isopropyl-β-D-thiogalactopyranoside (IPTG) (Sigma-Aldrich, St. Louis, MO, USA) to the growing culture, and the bacterial extract was pelleted and resuspended in phosphate-buffered saline (PBS). After induction, the cells were incubated for 5 h at 37°C with shaking at 200 rpm. The cells were harvested by centrifugation at 10,000 × g for 30 min at 4°C. The supernatant was discarded, and the cells were resuspended in lysis buffer (50 mM NaH_2_PO_4_, 20 mM imidazole, 300 mM NaCl, 1 mM PMSF, and 1× PLAAC) and lysed by extensive sonication (pulse on 4.4 s; pulse off 9.9 s; 60% extended for 2 min). The sample was centrifuged at 10,000 × g for 30 min at 4°C. His-tagged Pb14-3-3r was purified using a Ni-NTA column (GE Healthcare, Buckinghamshire, UK) equilibrated with 10 column volumes of buffer A (50 mM NaH_2_PO_4_, 20 mM imidazole, and 300 mM NaCl). Clarified lysate was applied to the column at a flow rate of 2–3 mL/min. The resin was washed with 5 column volumes of buffer A supplemented with increasing concentrations of imidazole (10–120 mM in 10 mM increments) followed by 10 column volumes of buffer A +250 mM imidazole. Eluted portions (10 mL) were collected from each imidazole concentration and analyzed by polyacrylamide gel electrophoresis (SDS-PAGE) [65]. The gels were washed, and the proteins were stained with Coomassie blue [66].

After obtaining the purified protein, the histidine tail was removed using the Thrombin Clean Cleave™ Kit (Sigma Aldrich, St. Louis, MO, USA) according to the manufacturer’s recommendations. The cleaved fractions were analyzed by SDS-PAGE.

To confirm that the purified recombinant protein obtained was indeed the 14-3-3 protein of *P. brasiliensis*, the bands obtained in the 10% polyacrylamide gel were purified and subjected to tryptic digestion using 10 ng/mL Trypsin Gold (Promega). The tryptic fragments were subjected to LC-MS/MS using a Cap-LC coupled to a Q-TOF Ultima API mass spectrometer (Waters, UK). The spectra were processed using ProteinLynx v4.0 software (Waters) and MASCOT MS/MS ion search (www.matrixscience.com), and the sequences were identified by searching the SwissProt database.

### Antibody Production

Purified recombinant 14-3-3 protein was used to generate specific rabbit polyclonal serum. Rabbit preimmune serum was obtained and stored at −20°C. The purified protein (1.5 mg/mL) was injected into one rabbit with Freund’s adjuvant three times at 2-week intervals. The obtained serum, containing monospecific polyclonal antibody to 14-3-3, was aliquoted and stored at −20°C. The immunoglobulin fractions of the antisera were separated by precipitation with ammonium sulfate and stored at −70°C.

### Cell-free Antigen

The cell-free extract was obtained from yeast cells of *P. brasiliensis* (isolate 18, with high adherence capacity to epithelial cells). The protein concentration of the extract was quantified by the Bradford method (BioRad), and the samples were analyzed by SDS-PAGE.

### Cell Wall Protein Extraction

This procedure was performed as described in da Silva Castro et al., [57], with some modifications. Yeast cells were frozen in liquid nitrogen and disrupted by maceration, and the material was lyophilized, weighed and resuspended in 50 mM Tris buffer. The supernatant was separated from the cell wall fraction by centrifugation at 10000 × g for 10 min at 4°C. To remove noncovalently linked proteins and intracellular contaminants, the isolated cell wall fraction was washed extensively with 1 M NaCl and boiled three times in SDS extraction buffer (50 mM Tris–HCl, pH 7.8, 2% w/v SDS, 100 mM Na–EDTA, and 40 mM β-mercaptoethanol) and pelleted after the extractions by centrifugation at 10000 × g for 10 min [67]. The protein concentration of the extract was quantified by the Bradford method (BioRad), and the samples were analyzed by SDS-PAGE.

### Western Blot Analysis

The cell wall protein extracts and purified 14-3-3 recombinant protein separated by one- and two-dimensional electrophoresis were transferred to nitrocellulose membranes. The membranes were incubated with the polyclonal antibody obtained against the 14-3-3 recombinant protein and peroxidase-conjugated anti-rabbit IgG as the secondary antibody. The reaction was developed with a chromogen substrate consisting of 0.005 g of diaminobenzidine (DAB) diluted in 30 mL of PBS plus 150 µL of hydrogen peroxide. The negative control reaction was performed with non-immune rabbit serum.

### Mice and IT Infection

C57BL/6 mice were obtained from the Isogenic Breeding Unit (Departmento de Imunologia, Instituto de Ciências Biomédicas, Universidade de São Paulo, São Paulo, Brazil) and used at 8 to 12 weeks of age. The mice were anesthetized and subjected to intratracheal (IT) *P. brasiliensis* infection as previously described [68]. Briefly, after intraperitoneal anesthesia, the animals were IT infected with 10^6^ *P. brasiliensis* yeast cells in 50 µL of PBS. At 72 h and 4 weeks postinfection, the lungs were removed and fixed to analyze the subcellular localization of *P. brasiliensis* 14-3-3 protein. These experiments were approved by the Ethics Committee on Animal Experiments of the University of São Paulo, São Paulo, Brazil.

### Subcellular Localization of the 14-3-3 Recombinant Protein in *P. brasiliensis* Yeast Cells *in vitro* and *in vivo*

To determine the subcellular localization of the 14-3-3 protein of *P. brasiliensis,* we performed immunocytochemistry at the ultrastructural level using immunogold labeling. For each experiment, both pneumocytes infected with *P. brasiliensis* (10^8^ cells/mL) for 2, 5 and 8 h and lungs removed from C57BL/6 mice IT infected with *P. brasiliensis* (10^6^ cells/mL) were fixed (2.5% v/v glutaraldehyde in 0.1 M sodium cacodylate buffer, pH 7.2) for 24 h at 4°C and submitted to the electron microscopy service of the Institute of Biomedical Sciences (ICB-I) USP-SP for the preparation of ultrathin sections. After fixation, the cells were rinsed several times using the same buffer, and free aldehyde groups were quenched with 50 mM ammonium chloride for 1 h, followed by block staining in a solution containing 2% (w/v) uranyl acetate in 15% (v/v) acetone for 2 h at 4°C (4). The material was dehydrated in a series of increasing concentrations of acetone (30 to 100% v/v) and embedded in LR Gold resin (Electron Microscopy Sciences, Washington, PA). The ultrathin sections were added to nickel grids, preincubated in 10 mM PBS containing 1.5% (w/v) bovine serum albumin (BSA) and 0.05% (v/v) Tween 20 (PBS-BSA-T), and subsequently incubated overnight with the polyclonal antibody against the 14-3-3 recombinant protein (diluted 1∶50). After washing with PBS-BSA-T, the grids were incubated overnight with the labeled secondary antibody (Au-conjugated rabbit IgG, 10 nm; diluted 1∶10). The controls were incubated with rabbit preimmune serum at 1∶50, followed by incubation with the labeled secondary antibody. After incubation, the grids were washed with the buffer described above, washed with distilled water, and stained with 3% uranyl acetate (w/v) and 4% lead citrate (w/v). Finally, the grids were observed with a Jeol 1010 transmission electron microscope (Jeol, Tokyo, Japan).

### Inhibition Assay of the Interaction between *P. brasiliensis* and Epithelial Cells Using Recombinant 14-3-3 Protein

The infection inhibition assays were performed on coverslips in 24-well plates. Pneumocyte monolayers (A549 cells) were cultured for approximately 24 h in Ham-F12 medium (Cultilab). Then, these monolayers were treated with 25 µg/mL of purified recombinant 14-3-3 protein for 1 h at 37°C. BSA was used as a control (25 µg/mL). At the indicated treatment times, the cells were washed and infected with 10^6^ cells/mL *P. brasiliensis* for 2 h, 5 h, 8 h and 24 h. Duplicates were analyzed in three independent experiments. After infection, the coverslips were washed and fixed with 4% paraformaldehyde for 1 h at room temperature. After fixation, the coverslips were stained with Giemsa and analyzed using an optical microscope. The number of fungi was counted in 5000 cells, and the total infection percentage was determined to determine the role of the 14-3-3 protein in the infection process. The data were confirmed by counting colony-forming units (CFUs). The test was also performed in 24-well plates without coverslips. After infection, the cells were washed, lysed with water and plated on Fava Netto’s medium supplemented with 4% fetal bovine serum. After 4 days, the CFUs were counted, and the data were statistically analyzed using Origin Pro v7.5 software.

### Inhibition Assay of the Interaction between *P. brasiliensis* and Epithelial Cells Using Polyclonal Anti-14-3-3 Produced in Rabbits

The infection inhibition assays were performed on coverslips in 24-wells plates. Pneumocytes monolayers (A549 cells) were cultured for in approximately 24 h in Ham-F12 medium (Cultilab). Then, suspensions of 10^6^ cells/ml of *P. brasiliensis* were pretreated with polyclonal anti-14-3-3 produced in rabbits (1∶100) and control with preimmune serum from rabbit 1∶100 in for 1 h at 37°C. At the indicated treatment times, the fungi were properly washed and this suspension was used to infect the epithelial cells. The times of infection were 2 h, 5 h, 8 h and 24 h. Duplicates were analyzed in three independent experiments. After the time of infection, the coverslips were washed and fixed with 4% paraformaldehyde for 1 h at room temperature. After fixation, the coverslips were stained with Giemsa and analyzed using an optical microscope. The number of fungi was counted in 5000 cells, and the total infection percentage was determinate to determine the role of 14-3-3 protein in the infection process. The data were confirmed by counting colony-forming units (CFUs). The test was also performed in the same way, but in 24-wells plates without coverslips. After the time of infection, the cells were washed, lysed with water and plated on Fava Nettós medium supplemented with 4% fetal bovine serum. After 4 days, the CFUs were counted, and data the were statistically analyzed using the Origin Pro v7.5 software.

## Results

### Homology of the Internal Peptides of the *P. brasiliensis* 30 kDa Adhesin

**Figure 1:**
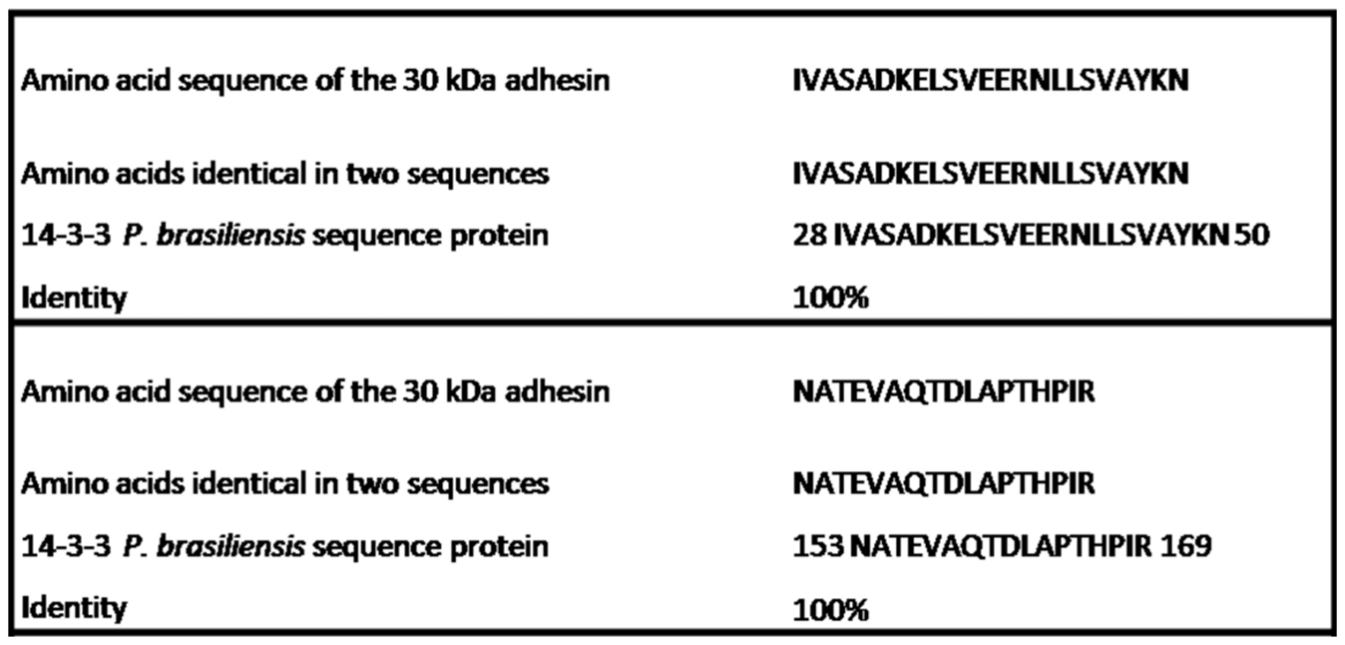
Comparison of the 30 kDa adhesin with the 14-3-3 protein of *P.brasiliensis* by BLASTp and FASTA 3.

The 30 kDa adhesin was analyzed based on sequences of the internal peptide of *P. brasiliensis*, which spanned three amino acid sequences: IVASADKELSVEER, NLLSVAYK and NATEVAQTDLAPTHPIR. These sequences were submitted to databases and analyzed by BLASTP (www.ncbi.nlm.nhi.gov/BLAST) and FASTA 3 (www.ebi.ac.uk/fasta33/). The results were the same in both analyses; the peptides shared similarity to the 14-3-3 protein of *P. brasiliensis*. The amino acid sequence of the peptides showed identity with two regions of the 14-3-3 protein of *P. brasiliensis* that were already deposited in GenBank (AAR24348): amino acids 28–50 shared 100% identity, and amino acids 153–169 shared 100% identity, as shown in Figure 1.

### Expression, Purification and Production of a Polyclonal Antibody to Pb14-3-3 Recombinant Protein

**Figure 2:**
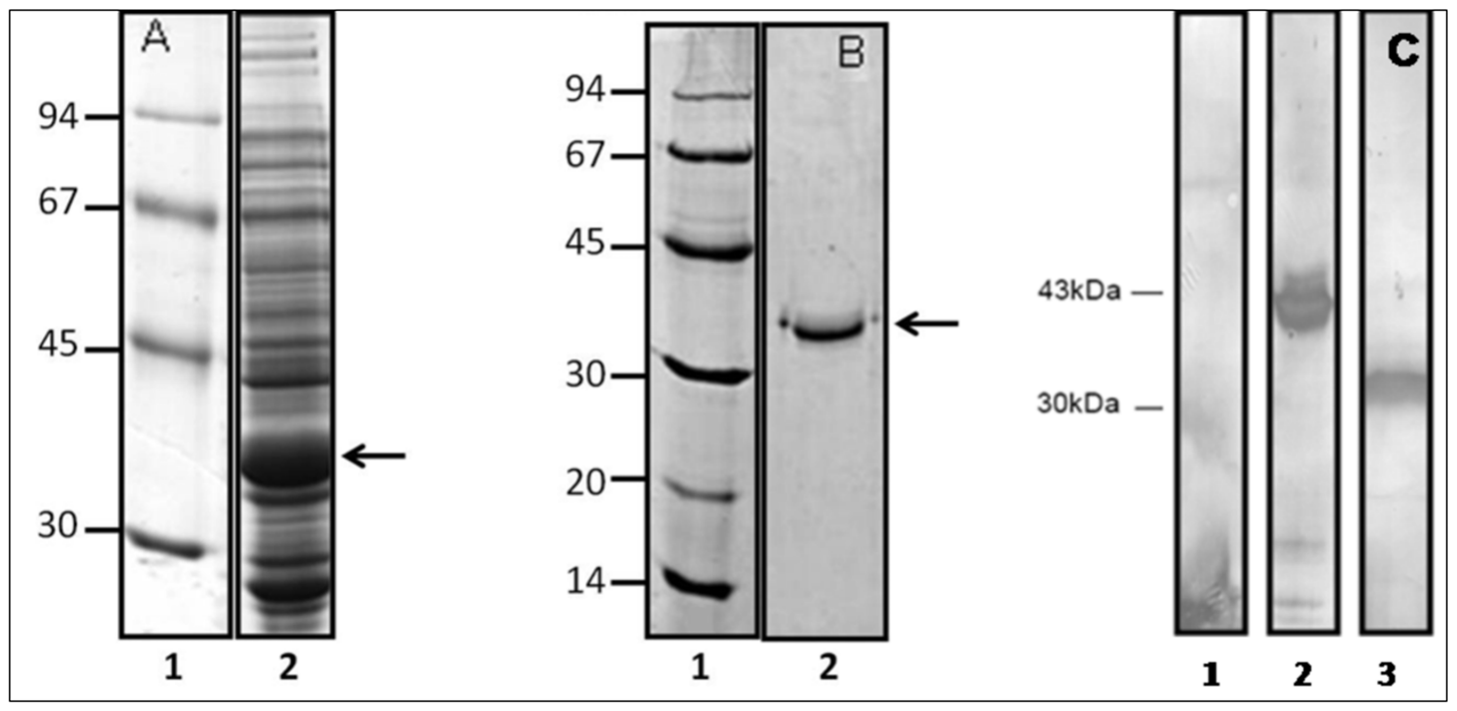
SDS-PAGE to verify protein induction (A) and 14-3-3 recombinant protein purification (B). Gels were stained with Phast Gel Coomassie R350. (A) 1: LMW ladder (low molecular weight, GE Life Science), 2∶5 h of induction with 0.4 mM IPTG at 37°C. (B) 1: LMW, 2: purified protein. The arrow indicates the purified 14-3-3 recombinant protein. (C) Immunoblot to verify the reactivity of polyclonal serum anti-14-3-3. (1) Pre-immune serum 1∶100– control, (2) full recombinant 14-3-3 protein and (3) cleaved 14-3-3 protein induced for 2 h.

cDNA encoding the Pb14-3-3 recombinant protein was subcloned into the expression vector pET-32a, and a recombinant fusion protein was obtained. After induction with IPTG, a 43 kDa recombinant protein was detected in bacterial lysates (Fig. 2A). The 6 histidine residues fused to the N-terminus of the recombinant protein were used to purify the protein from bacterial lysates through nickel-chelate affinity. The recombinant protein was eluted and analyzed by SDS-PAGE (Fig. 2B). An aliquot of the purified recombinant protein was used to generate a rabbit polyclonal antibody to Pb14-3-3r. Western blotting confirmed the positive reaction of the antibody with the fusion protein (Fig. 1C) and identified a 43 kDa protein in bacterial lysates. After cleavage with the Thrombin Cleave kit (Sigma Aldrich, St. Louis, MO, USA), the immunoreactive band corresponded to a 30 kDa protein. The 14-3-3 antiserum obtained in rabbits reacted with *P. brasiliensis* 14-3-3 recombinant protein, and reactivity was observed up to 1∶1000. Controls were incubated with rabbit preimmune serum at 1∶100 (Fig. 2C).

### Cell Wall Protein Extraction

**Figure 3:**
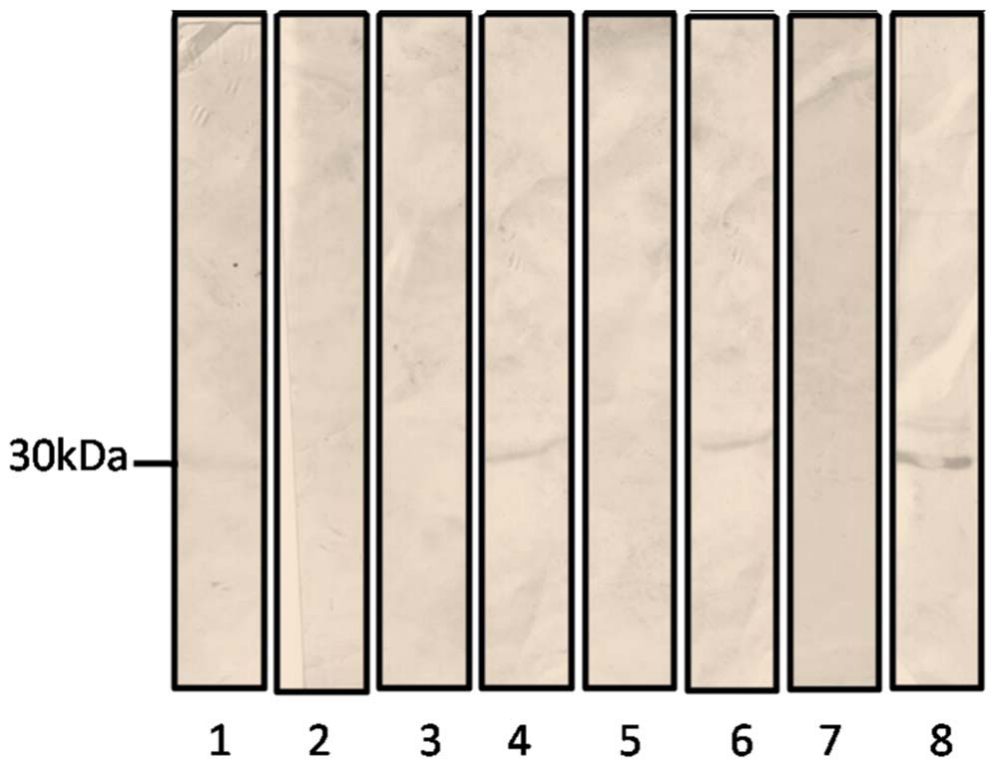
Immunoblotting was performed using the anti-Pb14-3-3 polyclonal antibody to verify 14-3-3 protein reactivity. Cytoplasmic fraction from *P. brasiliensis* grown in Fava Nettós media (1), cell wall fraction from *P. brasiliensis* grown in Fava Nettós media (2), and A549 cells infected with *P. brasiliensis* for 2 h (4), 5 h (6) and 8 h (8). The control was performed using noninfected A549 cells for 2 h (3), 5 h (5), and 8 h (7).

The 14-3-3 protein expression was more evident in *P.brasiliensis* cell wall recovered from A549 infected cells. When the fungus was cultivated in Fava Netto medium, we observed a weak reaction in the cytoplasmic fraction and no reaction in the cell wall extract (Fig. 3). Additionally, no reaction was observed in uninfected A549 cells (control) or in the cytoplasmic fraction of *P. brasiliensis* recovered from A549 infected cells (data not shown).

### Subcellular Localization of the 14-3-3 Protein in *P. brasiliensis* Yeast Cells *in vitro* and *in vivo*

**Figure 4:**
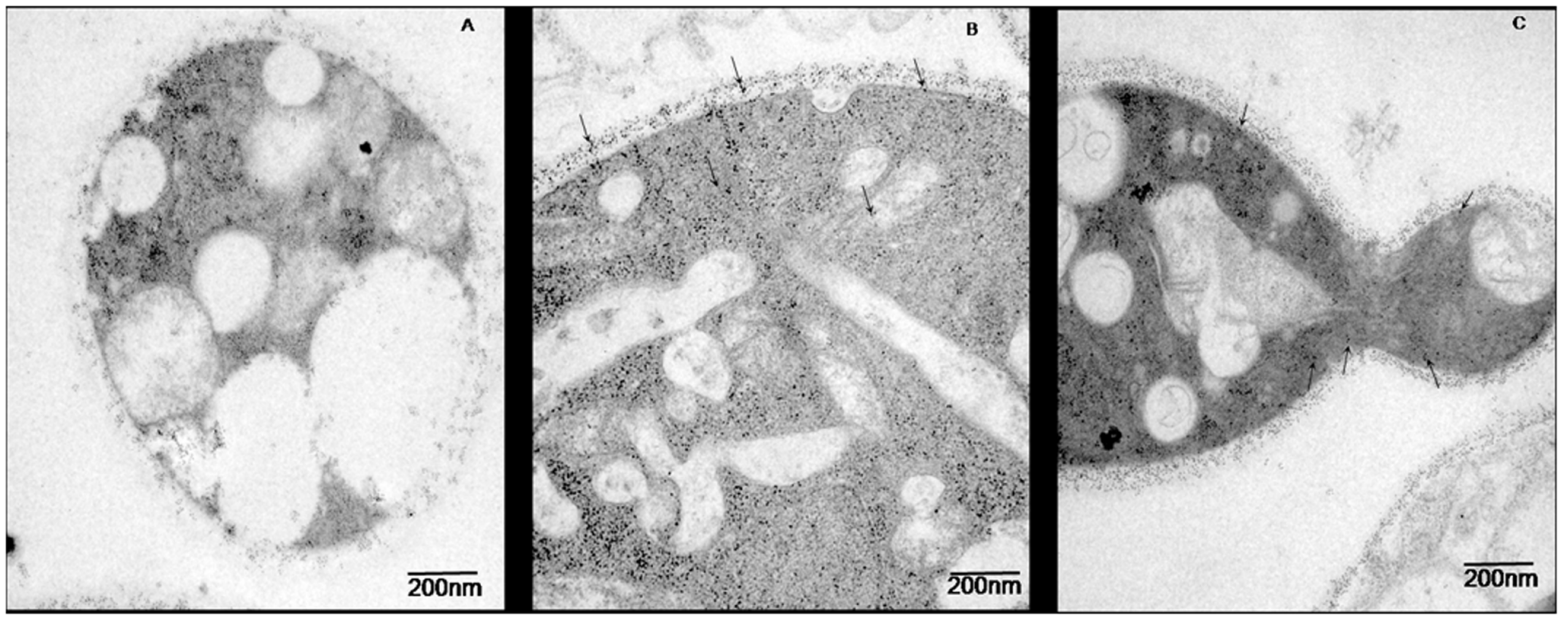
Immunoelectron microscopy to determine the localization of the 14-3-3 protein in yeast cells of *P. brasiliensis*. (A) The negative control was performed with pre-immune serum. (B) and (C) Labeling with the polyclonal anti-14-3-3 antibody. The arrows indicate the gold particles, demonstrating the sub-cellular localization of this protein. Magnification 25,000×.

The subcellular localization of the 14-3-3 protein was determined using an anti-14-3-3 polyclonal antibody in combination with immunoelectron microscopy. *P. brasiliensis* yeast cells, pneumocytes and lungs removed from C57BL/6 mice IT infected with *P. brasiliensis* were processed by postembedding with gold particles. Immunocytochemical assays revealed the ubiquitous distribution of gold particles in *P. brasiliensis* yeast cells, with some concentration in the cytoplasm (Fig. 4).

**Figure 5:**
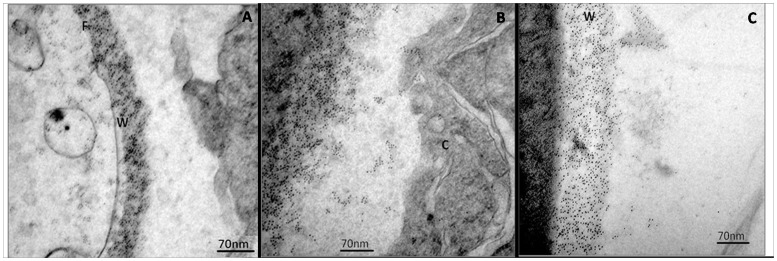
IEM detection of the 14-3-3 protein of *P.brasiliensis* during interaction with epithelial cells (A549) for 2 h (A), 5 h (B) and 8 h (C). Magnification 50,000×. F: fungus, W: fungal cell wall, V: vacuole, C: epithelial cell.

Notably, the number of gold particles was increased in the *P. brasiliensis* yeast cell wall at the time of epithelial cell interaction (Fig. 5), suggesting that the 14-3-3 protein may play an important role in the host-pathogen interaction. Some cell wall fragments containing these gold particles were directed to epithelial cells (Fig. 5C) at longer interaction times (8 h).

**Figure 6:**
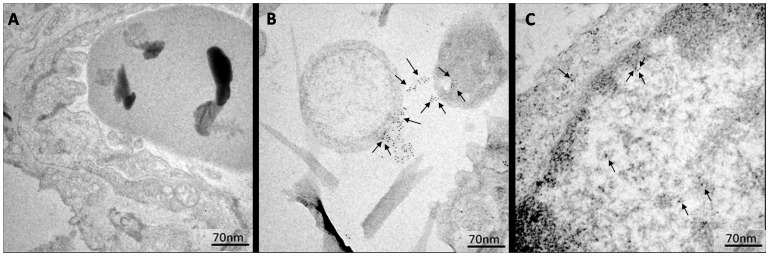
IEM detection of the 14-3-3 protein in *P.brasiliensis* yeast cells during acute mouse infection (B) and chronic mouse infection (C). Control: uninfected mice (A). The arrows indicate the gold particles, demonstrating the sub-cellular localization of this protein.

The 14-3-3 protein was ubiquitously distributed in fungi (Fig. 6) present at the sites of infection of C57BL/6 mice intratracheally infected with *P. brasiliensis* yeast cells for 72 h (acute infections) and 30 days (chronic infection).

Control samples incubated with rabbit preimmune serum showed no gold labeling.

### Inhibition of the Interaction of *P. brasiliensis* with Epithelial Cells Using Recombinant 14-3-3 Protein

**Figure 7:**
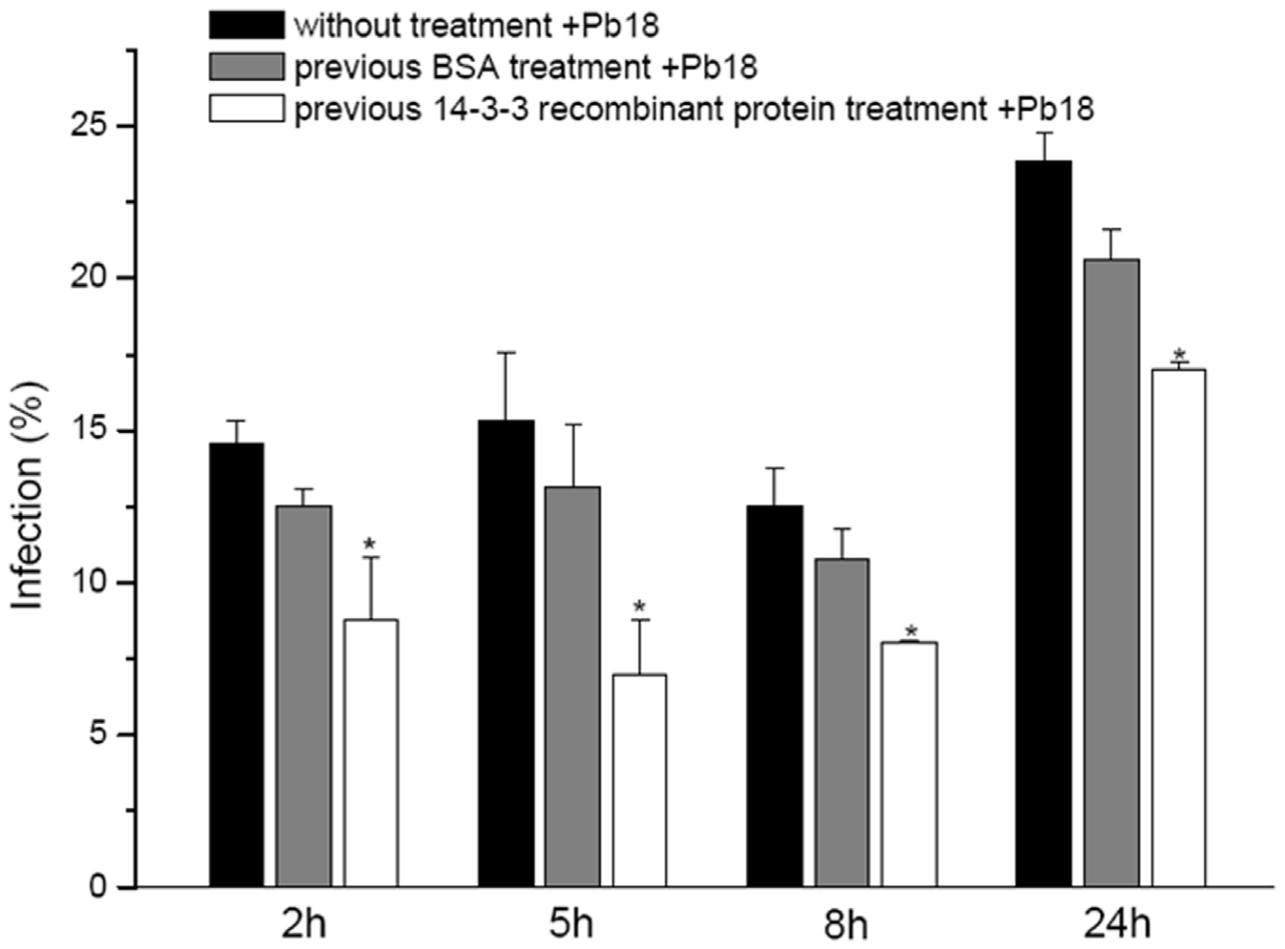
Inhibition assay of the interaction between *P.brasiliensis* and epithelial cells using recombinant 14-3-3 protein at different times. *p≤0.05 compared to untreated cells.

The inhibition assay was performed by counting cells using optical microscopy (Fig. 7). Pretreatment with the 14-3-3 protein of *P. brasiliensis* significantly reduced (p≤0.05) the infection at all times evaluated. These data were confirmed with CFU counts. BSA treatment (control) led to a slight reduction in the rate of infection, but these data were not statistically significant compared with those obtained in the absence of treatment. When the cells were pretreated with the recombinant 14-3-3 protein (25 µg/mL), we observed a reduction of approximately 40% at 2 h of infection, 54% at 5 h, 35% at 8 h and 28% at 24 h, demonstrating that this protein may be important in the *P. brasiliensis* infective process. Moreover, the rate of infection at 24 h was significantly different compared with earlier times (2, 5 and 8 h), but no difference was found between earlier times (p≤0.01). This result could explain the increased rate of infection, but there is still inhibition by recombinant 14-3-3 protein.

### Inhibition of the Interaction of *P. brasiliensis* with Epithelial Cells Using Polyclonal Anti-14-3-3 Produced in Rabbits

**Figure 8:**
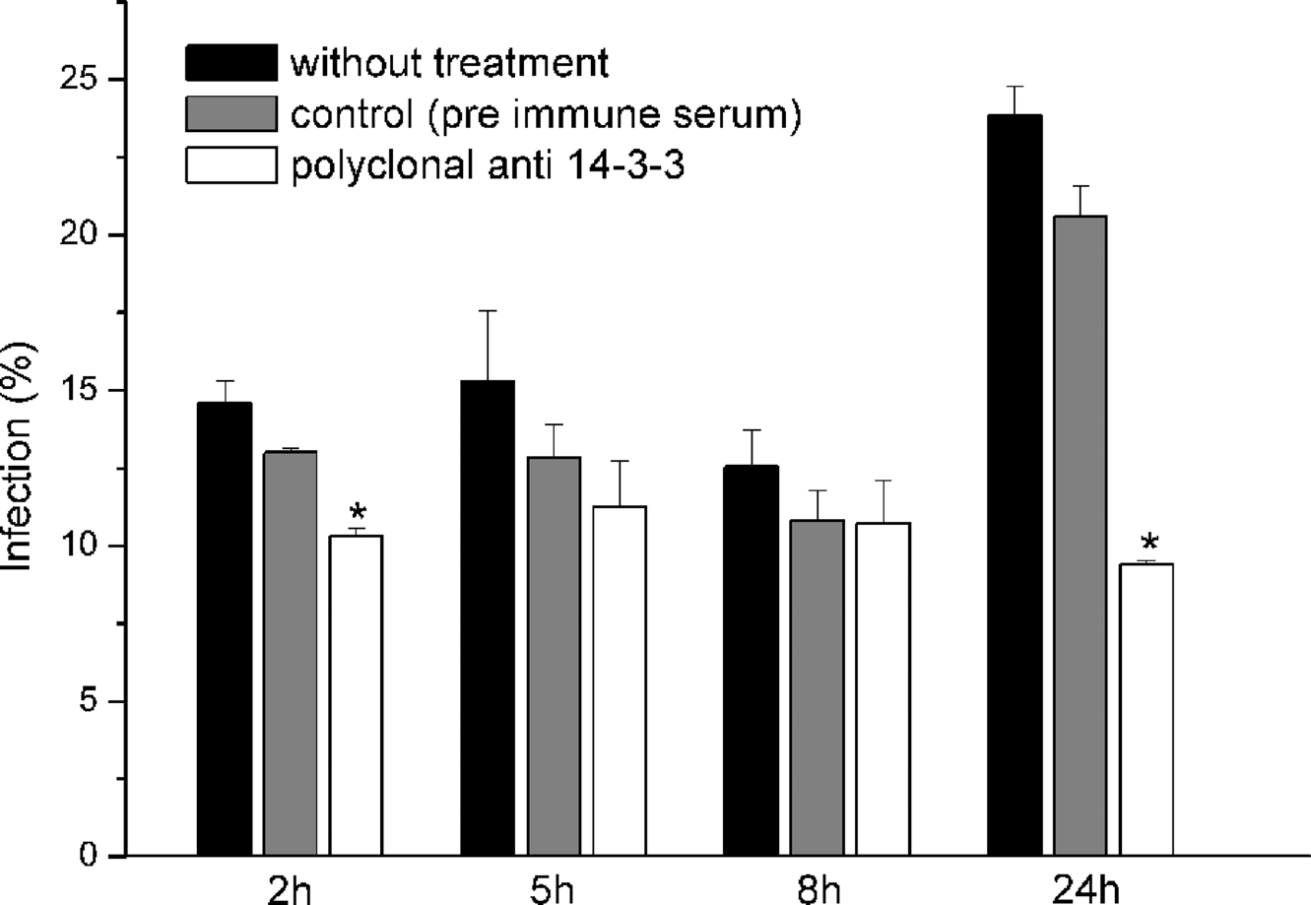
Inhibition of the interaction of ***P.*brasiliensis** with epithelial cells using polyclonal anti-14-3-3 produced in rabbits at different times. *p≤0.05 compared to untreated cells with cells infected with *P. brasiliensis* previously treated with polyclonal anti-14-3-3.

The inhibition assay was performed by counting cells using optical microscopy (Fig. 8). Antibody treatment (1∶100) was also effective in inhibiting the infection, particularly at 2 and 24 h, demonstrating that this protein may be important in the infective process of *P. brasiliensis*.

## Discussion

*P. brasiliensis* is considered a facultative intracellular fungus that may adhere to and invade epithelial cells *in vivo* and *in vitro* [2]. The adhesion and invasion ability of the fungus is dependent on the virulence of the isolate [3]. The ability of cells to interact with each other in an orderly manner depends on multiple adhesive interactions between adjacent cells and their extracellular environment and is mediated by cell adhesion molecules [69]–[71]. Pathogen adhesion requires the recognition of carbohydrate or protein ligands on the surface of the host cell or proteins of the ECM [20]–[22]. The large number of tissue types that fungi can colonize and infect suggests that fungi have a variety of surface molecules for adhesion [36]. Possible mechanisms responsible for determining the pathogenicity and virulence of *P. brasiliensis* have been extensively investigated by interaction experiments of this pathogen *ex vivo* in cell culture [26], [27], [37]–[42] and high-throughput molecular tools, such as cDNA microarrays, insertion and/or gene deletion, and RNA interference [14], [43]–[50].

In our previous study, we characterized a 30 kDa adhesin as a laminin ligand and observed that this adhesin was more highly expressed in virulent *P. brasiliensis* isolates, indicating that this protein may contribute to the virulence of this important fungal pathogen [39].

In the present study, we aimed to obtain a better understanding of the role of the 14-3-3 protein in the relationship between *P. brasiliensis* and host cells using *in vitro* and *in vivo* models. Thus, we generated a recombinant 14-3-3 protein in bacteria and used it to generate a polyclonal antibody that specifically recognized the recombinant purified protein.

Using amino acid sequencing, we determined that the adhesin belongs to the 14-3-3 family, and we showed that the *P. brasiliensis* protein may play an important role in the pathogenesis of this fungus, provided that inhibits by 50% the adherence to epithelial cells.

The 14-3-3 protein was identified in the genome of the *P. brasiliensis* fungus, but its function was unknown. The pathogen must regulate adhesin expression to survive and cause disease [39]. 14-3-3 proteins are a family of adaptor proteins that modulate protein function in all eukaryotic cells [72]. Little is known about the function of 14-3-3 proteins in pathogenic fungi.

Studies on *Saccharomyces cerevisiae* and *Schizosaccharomyces pombe* have demonstrated that both yeasts contain two genes that encode 14-3-3 proteins, and these proteins, as in higher eukaryotes, bind to numerous proteins involved in a variety of cellular processes [73]. The filamentous fungus *Aspergillus nidulans* contains a protein with high homology to 14-3-3 proteins (called Arta) that prevents the formation of the septum [74], and recently, this protein was described in *P. brasiliensis* vesicles [19], [75].

A critical first step in the establishment of infection by pathogens is adhesion to host components. The recognition of host cells by a pathogen requires the presence of complementary molecules on the surface of the host cell. We previously demonstrated that *P. brasiliensis* is capable of adhering to and invading epithelial cells [27]. Adhesins that interact with receptors have been found to exist in a number of different pathogens, and host components of the ECM are often of great importance in the modulation of migration, invasion, differentiation, and microbial proliferation. In recent years, several proteins in *P. brasiliensis* with receptor-like characteristics have been found to be ligands of the ECM [26], [27], [33], [35], [39], [76].

Using enteropathogenic *E. coli* (EPEC), Patel et al., (2006) demonstrated that the tau isoform (also known as theta) of 14-3-3 can bind specifically to Tir, a major effector protein that is delivered to the plasma membrane of the eukaryotic cell, where it acts as the receptor for the bacterial adhesin intiminin. 14-3-3tau is recruited to the site of the pedestal (3 h after infection) and can decorate attached EPEC in the later stages of the infection process (5–7 h after infection) [72].

Immunocytochemical analysis, confirmed by western blotting analysis of cell wall protein extracts, revealed ubiquitous distribution of the 14-3-3 protein in the cell wall of the yeast form of *P. brasiliensis,* with some concentration in the cytoplasm, and in *in vitro* (pneumocyte interaction) and *in vivo* (mouse infection) models. Interaction experiments were also carried out in animal models of infection (C57BL/6 mice) to elucidate the role of this protein *in vivo* and validate the data previously obtained in cell culture. Notably, we observed a large increase in the amount of *P. brasiliensis* 14-3-3 protein in the fungal cell wall during interaction with epithelial lung cells (A549) and in acute infection in mice, suggesting that this protein could play an important role in the host-pathogen interaction. Few fungi are found in acute infections; however, we observed the presence of the 14-3-3 protein in the fungal cell wall and a partial loss of this cell wall, similar to the cell culture model (A549). However, in chronic infection (30 days), the distribution of the 14-3-3 protein was similar to that found in fungus in culture media, and this feature may be related to an adaptive condition of the fungus. The 14-3-3 protein distribution in *P. brasiliensis* during the interaction with epithelial lung cells and in infected mice has never been demonstrated, and the large amount of 14-3-3 protein in the cell wall of this fungus during the interaction may suggest the importance of this protein in this context.

The presence of the 14-3-3 protein at the *P. brasiliensis* cell surface raises interesting questions: for example, how is this protein incorporated into the cell wall in the absence of a conventional N-terminal signal sequence for targeting the protein to the secretory pathway. Additional studies will be necessary to identify putative signals related to *P. brasiliensis* cell wall targeting. The targeting of some classic cytoplasmic molecules lacking an N-terminal signal peptide to other cellular compartments is not uncommon for *P. brasiliensis*, as described for glyceraldehyde-3-phosphate-dehydrogenase (GAPDH) and triosephosphate isomerase (TPI) [34]. Proteins that lack an N-terminal signal peptide sequence have also been found in the cell wall of *S. cerevisiae* in addition to their usual cytoplasmic localization [77]. In addition, the cytoplasmic proteins GAPDH, TPI and formamidase have been detected in extracellular vesicles secreted by *Histoplasma capsulatum* [78] and *Cryptococcus neoformans* [79]. These data support our finding that the 14-3-3 protein is localized in both the cytoplasm and the cell wall of *P. brasiliensis* [75], and during the interaction, it can be exported to sites of infection.

In conclusion, in the present study, we have shown that the *P. brasiliensis* 14-3-3 protein, with adhesin characteristics, may play an important role in the fungus-host cell interaction. Our data may lead to a better understanding of *P. brasiliensis* interactions with host tissues and paracoccidioidomycosis pathogenesis.
