# Correction: Paracoccidoides brasiliensis 30 kDa Adhesin: Identification as a 14-3-3 Protein, Cloning and Subcellular Localization in Infection Models

**DOI:** 10.1371/journal.pone.0142028

**Published:** 2015-10-30

**Authors:** Julhiany de Fatima da Silva, Haroldo César de Oliveira, Caroline Maria Marcos, Rosângela Aparecida Moraes da Silva, Tania Alves da Costa, Vera Lucia García Calich, Ana Marisa Fusco Almeida, Maria José Soares Mendes-Giannini

In Fig 3, panel 6 is a mistaken duplication of panel 4.The authors have provided a corrected version of Fig 3 here that includes a new image for panel 6. The original blots used to create the revised panel and figure as well as original blots for both the cytoplasmic fraction and the cell free extract can be viewed in S1 Data. The authors confirm that this error does not alter their results.

**Fig 3:**
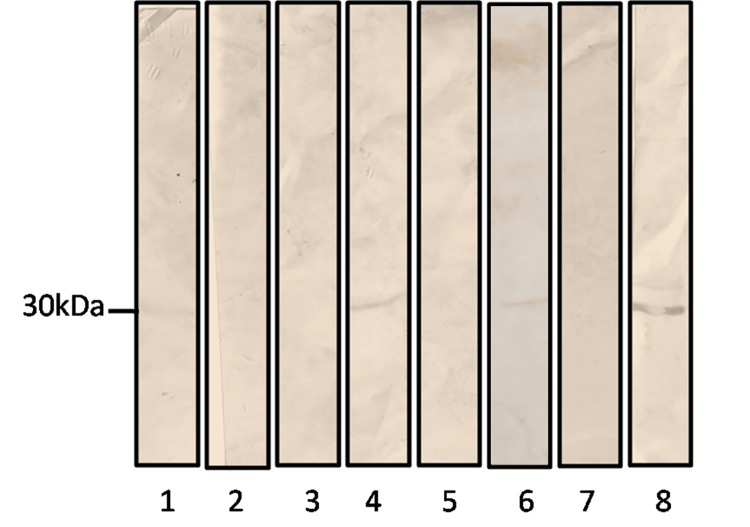
Immunoblotting was performed using the anti-Pb14-3-3 polyclonal antibody to verify 14-3-3 protein reactivity. Cytoplasmic fraction from P. brasiliensis grown in Fava Nettós media (1), cell wall fraction from P. brasiliensis grown in Fava Nettós media (2), and A549 cells infected with P. brasiliensis for 2 h (4), 5 h (6) and 8 h (8). The control was performed using noninfected A549 cells for 2 h (3), 5 h (5), and 8 h (7).

S1 Data Original blots for cell well extract, cell free extract, and cytoplasmic fraction. (ZIP)
