# Supplementary figures and images for: Correction: Paracoccidoides brasiliensis 30 kDa Adhesin: Identification as a 14-3-3 Protein, Cloning and Subcellular Localization in Infection Models
Source: PLoS One. 2015 Oct 30;10(10):e0142028. doi: 10.1371/journal.pone.0142028 (PMC4627770; doi:10.1371/journal.pone.0142028)

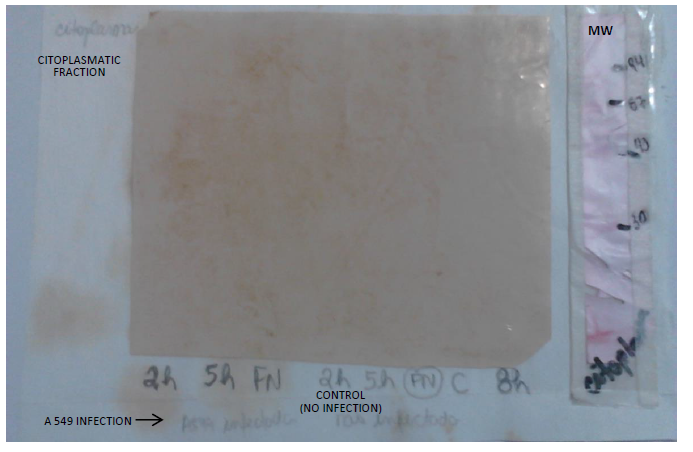

Supplement: S1 Data — (ZIP) [file pone.0142028.s001.zip › Figure C erratum PONE 14-3-3 600dpi (1).tif]

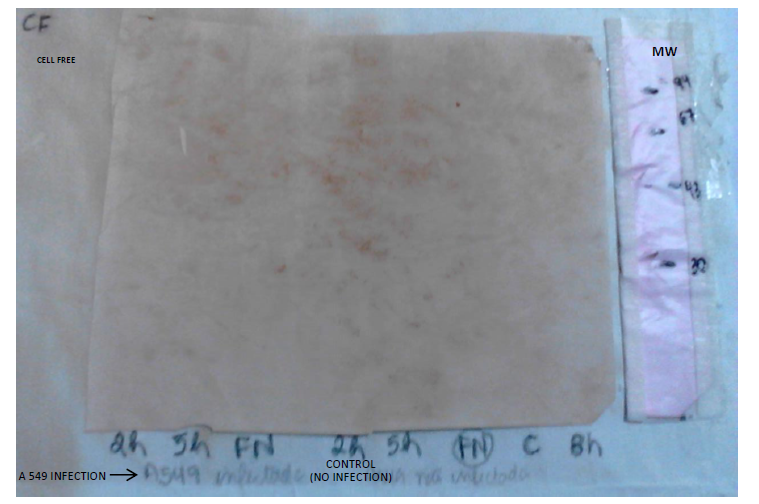

Supplement: S1 Data — (ZIP) [file pone.0142028.s001.zip › Figure B erratum PONE 14-3-3 600dpi.tif]

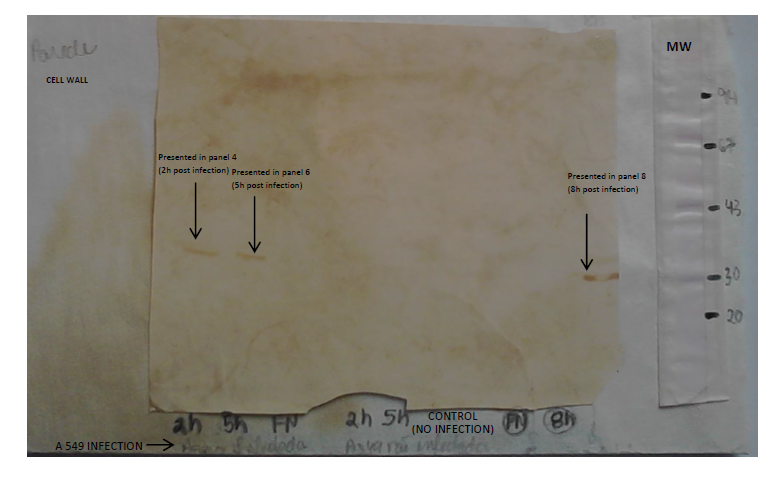

Supplement: S1 Data — (ZIP) [file pone.0142028.s001.zip › Figure A erratum PONE 14-3-3 600dpi (1).tif]
